# Supplementary material for: Inflammatory factors and risk of lung adenocarcinoma: a Mendelian randomization study mediated by blood metabolites
Source: Front Endocrinol (Lausanne). 2024 Aug 27;15:1446863. doi: 10.3389/fendo.2024.1446863 (PMC11384989; doi:10.3389/fendo.2024.1446863)
Supplement: Supplementary file 1 [file DataSheet1.zip › supplementary files/S2/GCST90200409/sensitivity-analysis.pdf]

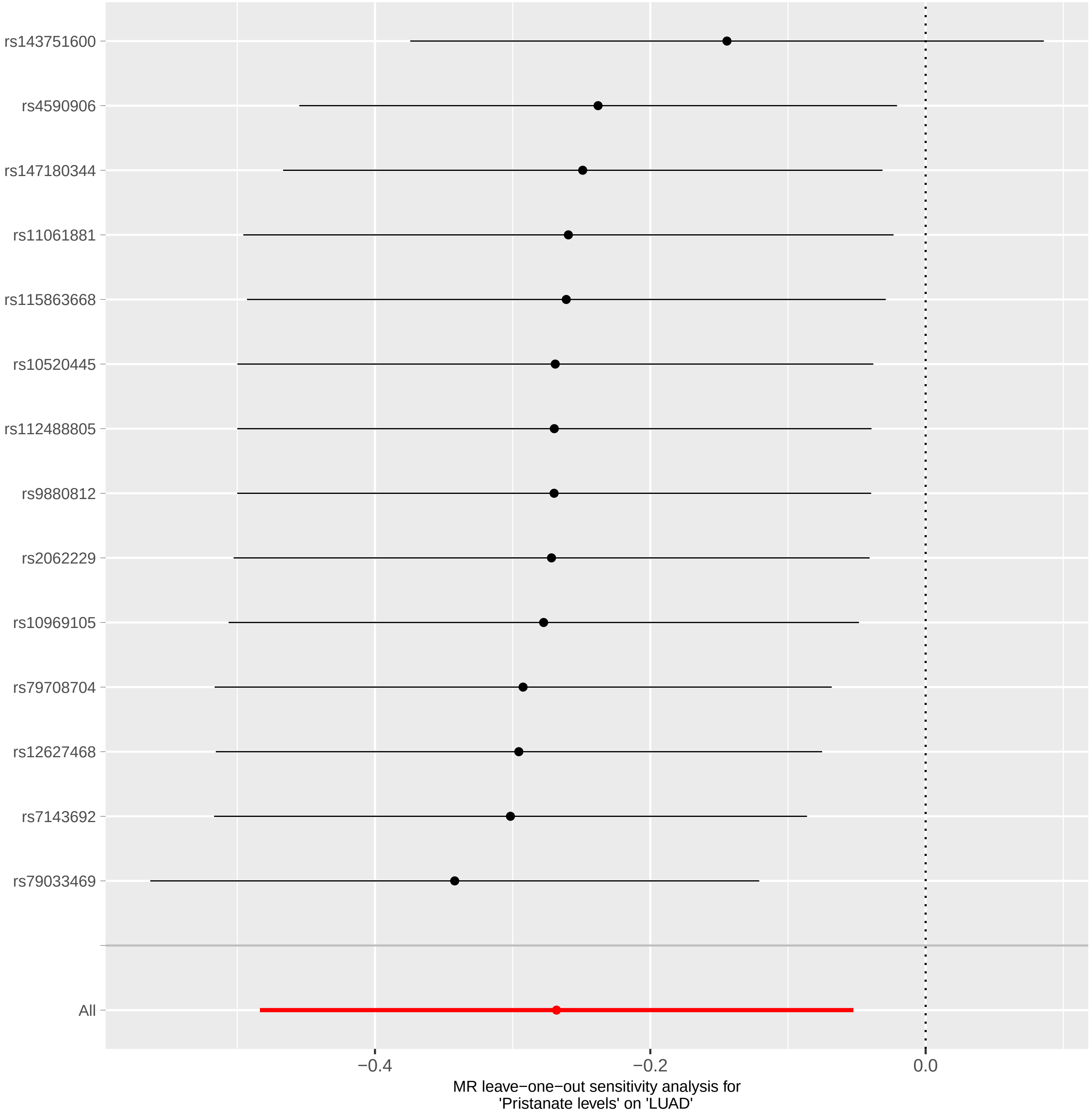
rs143751600
rs4590906
rs147180344
rs11061881
rs115863668
rs10520445
rs112488805
rs9880812
rs2062229
rs10969105
rs79708704
rs12627468
rs7143692
rs79033469
All
−0.4
−0.2
0.0
MR leave−one−out sensitivity analysis for
'Pristanate levels' on 'LUAD'
